# Crowded living and its association with mental ill-health among recently-arrived migrants in Sweden: a quantitative study

**DOI:** 10.1186/s13104-018-3718-6

**Published:** 2018-08-24

**Authors:** Elisabeth Mangrio, Slobodan Zdravkovic

**Affiliations:** 1 Department of Care Science, Faculty of Health and Society, Malmö University, Malmö, Sweden; 2 Malmö Institute for Studies of Migration, Diversity and Welfare (MIM), Malmö University, Malmö, Sweden

**Keywords:** Migration, Crowded living, Mental ill-health

## Abstract

**Objective:**

Housing and neighbourhood conditions are widely acknowledged important social determinants of health and health inequalities that persist in developed countries despite general improvements in health outcomes across populations. Previous research has investigated what effect crowded living conditions have on mental health and concluded that women living in crowded conditions were more likely to suffer from depression. In contrast, men living in the same conditions responded with withdrawal or aggression. To the best of our knowledge, only a few studies have examined the association between recently-arrived migrants living in crowded conditions and poor mental health. The aim of this study was to investigate the association between crowded living conditions among recently-arrived migrants in Sweden and mental ill-health. The result is based on 681 migrants who completed and returned questionnaires in 2015–2016.

**Results:**

The analyses, independent of gender, resulted in a significant unadjusted odds ratio of 1.46 (95% CI 1.05–2.03); even after adjustments were made, the association remained significant OR 1.47 (1.05–2.07). When adding stability in housing into the adjustment-model, the OR did not remain significant OR 1.40 (0.99–1.99), P-value 0.061.

## Introduction

Migrants’ mental health issues frequently present a challenge to clinicians and policy makers in the recipient country [1]. The mental health of migrants may by influenced by the migration experience, and some scholars define this as ‘pre-migration trauma and post-migration living difficulties’ [2]. Some studies report a gradual improvement in symptoms over a period of a decade, to the point where the observed prevalence rates of mental disorder become lower than that of the general population of the host country [3, 4]. Other studies have found prevalence rates that were substantially higher than those of the general population [5–7]. The different outcome regarding mental health among migrants could according to Hansson et al. [8] depend on differences in social context as well as country of origin, age and socioeconomic status. These factors could affect mental health for everyone in the population but migrants could be more exposed to the negative effects of these determinants [8].

Housing and neighbourhood conditions are widely acknowledged to be important social determinants of health [9] and health inequalities persisting in developed countries despite general improvements in health outcomes across the population [10]. Evans et al. suggest that various characteristics of housing quality may influence psychosocial processes, which in turn can affect mental health [11]. According to Krieger & Higgins, crowded living has been associated with psychological stress among women aged 25–45 in London as well as living with temporary housing, has been shown to cause behavioural problems among children [12]. Another study by Gove et al. [13], suggest as well that crowded living could affect mental health negatively. In an American study, the authors noticed that mothers that experienced housing disarray and housing instability were more likely to screen positive for depression [14]. In another American study by Evans et al. [15], an association was seen between children living in crowded conditions and effects on their psychological health. This concerned children living in both urban and rural areas. Furthermore, women experiencing crowded living conditions were more likely to suffer from depression than men experiencing the same, with the latter likely to suffer from withdrawal or aggression [16]. Since previous research indicates that housing conditions could be associated with mental ill-health [11–14] as well as that recently-arrived migrants (RAM) suffer from mental ill-health [1, 2, 5–7], it is of great importance to investigate if crowded living could affect mental ill-health among RAM in Sweden. The aim of this study was, therefore, to investigate the association between crowded living conditions among RAM in Sweden and mental ill-health.

## Main text

### Material and methods

Approximately 1700 questionnaires were distributed to RAM speaking Arabic or Dari and who participated in the mandatory public integration support programme in the Scania region of Sweden. The study population was recruited by inviting the participation of all eligible adult migrants who, between February 2015 and February 2016, were taking part in the compulsory civic and health information programme as part of the introduction plan for all RAM in Sweden [17]. In total, 681 questionnaires were returned, resulting in a response rate of approximately 39.5%. The vast majority of the study’s participants were granted their residence permit as asylum seekers or as part of family reunification; for the purpose of this research, they are considered as refugees. They have all fled from war-zones such as Syria, Iraq and Afghanistan. They were housed either in accommodations supplied by the government or in apartments arranged by themselves. Data collection was assessed by a self-administered questionnaire that included questions about health, sleep, level of education, well-being, accommodation type, social relations, work and access to health care.

#### Dependent variable

Mental health was assessed by the General Health Questionnaire (GHQ-12) scale [18], which is a combination of 12 questions regarding a person’s well-being and sleep. The questions ask for information about sleep, stress, concentration, decision-making, tension, problem-solving, sadness and depression. These questions were assessed together and responses were grouped into two categories: good metal health and poor mental health. If three or more of the twelve items indicated poor psychological health, the respondent’s GHQ-12 was defined as poor.

#### Independent variables

Crowded living was considered as the main exposure derived from the following questions: How many persons live in your house? How many rooms do you have? If more than two persons were living in the same bedroom, the housing was considered as crowded. This variable was based on a report from Statistics Sweden [19] and this way of calculating crowded living has been done in other Swedish reports as well [20].

Housing condition responses were categorised as indicating good living conditions if the person reported (a) living in a rental flat without a creditor or (b) owning an apartment. Unstable housing conditions were defined as (a) living in a rental flat with a creditor, (b) having a sub-let apartment, (c) living with friends or family or (d) living in a government accommodation facility.

Educational level was based on years of schooling, divided into low educational level (9 years or less), medium educational level (10–12 years of school) and high educational level (more than 12 years).

Age was measured as a continuous variable; but for descriptive statistics, it was divided into five categories: aged 18–34, 35–44, 45–54, 55–64 and 65–80.

Gender was divided into either male or female.

#### Statistical analysis

Descriptive statistics were calculated as frequencies and percentages. Logistic regression was used to analyse the association between the dependent variable mental health and the independent variable crowded living by calculating odds ratios, P-values and 95% confidence intervals. Multiple logistic regression was used to adjust the estimated odds ratios for the influence of confounding factors such as educational level, age and housing condition. The choice of variables in the adjustment model was based on previous research [21]. Statistical analyses were performed by SPSS version 22.

## Results

The study included 461 males, 204 females and 16 who did not specify their gender. There were 307 respondents between the ages of 18 and 34 and 155 between the ages of 35 and 44. A total of 301 respondents had a high educational level (218 males and 83 females), 141 had a medium educational level (94 males and 47 females) and 146 had a low educational level (102 males and 44 females). Of the 681 respondents, 166 rented an apartment without a creditor (111 males and 55 females) and 198 were living with friends or family (152 males and 46 females).

The analyses performed, independent of gender, resulted in a significant odds ratio of 1.46 (95% CI 1.05–2.03), see Table 1. Stratifying by gender resulted in non-significant OR for both men 1.32 (0.89–1.96) and women 1.84 (1.0–3.40). Although, the odds ratio for women was borderline significant with the P-value of 0.051.

**Table 1:** Crowded living and the association with mental ill-health

Living conditions	Males		Females		All	
	Number (%)	OR* 95%(CI) P-value	Number (%)	OR* 95%(CI) P-value	Number (%)	OR* 95% (CI) P-value
No crowded living	187 (47.2)	1	89 (53)	1	276 (48.9)	1
Crowded living	209 (52.8)	1.32 (0.89–1.96) 0.168	79 (47)	1.84 (1.0–3.40) 0.051	288 (51.1)	1.46 (1.05–2.03) 0.026

Adjusting for age and educational level resulted in a non-significant OR for men 1.06 (0.94–1.2) and for women 1.01 (0.84–1.22). However, when considering both women and men together, the OR became significant 1.47 (1.05–2.07), see Table 2. When adding stability in housing into the adjustment-model, the results turned out to be non-significant OR 1.40 (0.99–1.99), see Table 3.

**Table 2:** Crowded living and the association with mental ill-health, adjusted for age and educational level

Living conditions	Males		Females		All	
	Number (%)	OR* 95%(CI) P-value	Number (%)	OR* 95%(CI) P-value	Number (%)	OR* 95%(CI) P-value
No crowded living	187 (47.2)	1	89 (53)	1	276 (48.9)	1
Crowded living	209 (52.8)	1.06 (0.94–1.2) 0.346	79 (47)	1.01 (0.84–1.22) 0.887	288 (51.1)	1.47 (1.05–2.07) 0.026

**Table 3:** Crowded living and the association with mental ill-health, adjusted for age, educational level and stability in housing

Living conditions	All	
	Number (%)	OR* 95%(CI) P-value
No crowded living	276 (48.9)	1
Crowded living	288 (51.1)	1.40 (0.99–1.99) 0.061

## Discussion

The results showed that being a RAM and living in crowded living conditions increased the odds for suffering from mental ill-health, if the whole population was analysed both before and after adjustment were made for age and educational level. However, after adjusting the results for stability of housing, the results turned non-significant. The results after adjustments for stability of housing was borderline-significant, which indicates that crowded living could have a small effect on mental-ill-health, but to a greater extent is stability of housing effecting mental ill-health OR 1.40 (0.99–1.99). This is in line with the results from the American study by Suglia et al. [14], which saw that insecurity in housing increased the odds for mothers suffering from depression. In Sweden, there is currently a lack of apartments with an affordable rent, which makes it difficult for RAM to find a stable housing condition [22]. Efforts are taken for finding improvement regarding this concern [22]. This is important since we know that RAM could be at a higher risk of depression, psychosis and suicidal thoughts [23], and since we know that participation in the establishment process for RAM requires active participation, it is of great importance to reduce the risk for increased suffering from mental ill-health, as well as reducing the risk for those unable to establish themselves successfully in a new country. When considering both the results from the current study, as well as those mentioned in the earlier studies concerning housing conditions [11–14], it is of great importance to work for a suitable and stable housing for the RAM in Sweden.

## Limitations

The strength of the current study is that it targets health issues as closed to the arrival of RAM to Sweden as possible. As compared to the latest regional public health survey in Scania, the current response rate is lower [20, 24]. However, when comparing the response rate of both studies—more precisely, respondents with a country of birth outside of Europe—the current response rate of 39.5% is moderately higher. Furthermore, the response rate is about the same or higher than that of other studies in the same field [20, 24]. The response rate resulted in 681 study participants, which probably is the reason why the gender specific analyses turned out to be non-significant after adjustments for age and educational level. When adjustments were made for stability of housing, the analysis could only be made with men and women together, due to too less power.

GHQ-12 was used as a tool for assessing mental health and this instrument is internationally well validated for assessing mental ill-health [18, 25]. This instrument is the shortest of the GHQ measuring instruments, but has shown itself to be a secure measure of psychological health [18] and its validity characteristics are not influenced by gender, age or educational level [18]. The GHQ-12 scale has shown good psychometric properties in other studies [26–28]. Crowded living was defined as crowded on basis of a definition from Statistics Sweden [19] and in Sweden, there are three ways to calculate crowded living [19] and we have selected the version already specified. It could be discussed how well this definition is well-suited for refugees that could have lived with other cultural practices regarding living conditions. However, this is a definition that is based on two people per room compared to the other two definitions with one or three persons per room [19] and we considered this version as a mean version between the two other mentioned.

The fact that all participants were anonymous made any dropout analyses impossible. However, an approximate dropout analysis was performed by comparing the characteristics of the study participants with statistics from Swedish Public Employment Service. The analysis suggested that people with higher levels of education were over-represented in the present study [29]. It would have been considered beneficial if a greater population of migrants could have been investigated. However, it was only possible to follow these migrants on a regional level, although a national level would be better.

## References

[1] Bogic M, Njoku A, Priebe S (2015). Long-term mental health of war-refugees: a systematic literature review. BMC Int Health Hum Rights.

[2] Kirmayer LJ, Narasiah L, Munoz M, Rashid M, Ryder AG, Guzder J, Hassan G, Rousseau C, Pottie K (2011). Canadian collaboration for immigrant and refugee health (CCIRH): common mental health problems in immigrants and refugees: general approach in primary care. CMAJ.

[3] Beiser M, Hou F (2001). Language acquisition, unemployment and depressive disorder among Southeast Asian refugees: a 10-year study. Soc Sci Med.

[4] Silove D, Steel Z, Bauman A, Chey T, McFarlane A (2007). Trauma, PTSD and the longer-term mental health burden amongst Vietnamese refugees. Soc Psychiatry Psychiatr Epidemiol.

[5] Carlson EB, Rosser-Hogan R (1994). Cross-cultural response to trauma: a study of traumatic experiences and posttraumatic symptoms in Cambodian refugees. J Trauma Stress.

[6] D’Avanzo CE, Barab SA (1998). Depression and anxiety among Cambodian refugee women in France and the United States. Issues Ment Health Nurs.

[7] Marshall GN, Schell TL, Elliott MN, Berthold SM, Chun C (2005). Mental health of Cambodian refugees 2 decades after resettlement in the United States. JAMA.

[8] Hansson EK, Tuck A, Lurie S, McKenzie K (2012). Rates of mental illness and suicidality in immigrant, refugee, ethnocultural, and racialized groups in Canada: a review of the literature. Can J Psychiatry.

[9] Gibson M, Petticrew M, Bambra C, Sowden AJ, Wright KE, Whitehead M (2011). Housing and health inequalities: a synthesis of systematic reviews of interventions aimed at different pathways linking housing and health. Health Place.

[10] Health inequalities—progress and next steps. http://webarchive.nationalarchives.gov.uk/20130123194028/http://www.dh.gov.uk/en/Publicationsandstatistics/Publications/PublicationsPolicyAndGuidance/DH_085307. Accessed 22 Aug 2018.

[11] Evans GW, Wells NM, Moch A (2003). Housing and mental health: a review of the evidence and a methodological and conceptual critique. J Soc Iss.

[12] Krieger J, Higgins DL (2002). Housing and health: time again for public health action. Am J Public Health.

[13] Gove WR, Hughes M, Galle OR (1983). Overcrowding in the household: an analysis of determinants and effects.

[14] Suglia SF, Duarte CS, Sandel MT (2011). Housing quality, housing instability, and maternal mental health. J Urban Health.

[15] Evans GW, Saegert S, Harris R (2001). Residential density and psychological health among children in low-income families. Environ Behav.

[16] Regoeczi WC (2008). Crowding in context: an examination of the differential responses of men and women to high-density living environments∗. J Health Soc Behav.

[17] Björngren Cuadra C, Carlzen K (2015). MILSA-support platform for migration and health. Laying the foundation.

[18] Goldberg DP, Gater R, Sartorius N, Ustun TB, Piccinelli M, Gureje O, Rutter C (1997). The validity of two versions of the GHQ in the WHO study of mental illness in general health care. Psychol Med.

[19] Statistics Sweden: Var finns rum för våra barn? En rapport om trångboddhet i Sverige; 2006. **(In Swedish)**. https://www.boverket.se/globalassets/publikationer/dokument/2006/var_finns_rum-for_vara_-barn.pdf. Accessed 22 Aug 2018.

[20] Fridh M, Birgit M, Lindström M, Grahn M, Rosvall M: Folkhälsorapport Skåne 2013-en undersökning om vuxnas livsvillkor, levandsvanor och hälsa. Malmö, Sweden: Region Skåne; 2013. **(In Swedish)**. https://utveckling.skane.se/siteassets/publikationer_dokument/folkhalsorapport_skane_2013.pdf. Accessed 22 Aug 2018.

[21] Bonita R, Beaglehole R, Kjellström T (2006). Basic epidemiology.

[22] https://www.boverket.se/sv/samhallsplanering/bostadsplanering/bostadsmarknaden/bostadsmarknadsenkaten-i-korthet/olika-grupper/nyanlanda/. Accessed 22 Aug 2018.

[23] Gilliver SC, Sundquist J, Li X, Sundquist K (2014). Recent research on the mental health of immigrants to Sweden: a literature review. Eur J Public Health.

[24] Christensen AI, Ekholm O, Glümer C, Andreasen AH, Hvidberg MF, Kristensen PL, Larsen FB, Ortiz B, Juel K (2012). The Danish national health survey 2010. Study design and respondent characteristics. Scand J Soc Med.

[25] McDowell I (2006). Measuring health: a guide to rating scales and questionnaires.

[26] Gelaye B, Tadesse MG, Lohsoonthorn V, Lertmeharit S, Pensuksan WC, Sanchez SE, Lemma S, Berhane Y, Vélez JC, Barbosa C (2015). Psychometric properties and factor structure of the General Health Questionnaire as a screening tool for anxiety and depressive symptoms in a multi-national study of young adults. J Affect Disord.

[27] Fernandes HM, Vasconcelos-Raposo J (2013). Factorial validity and invariance of the GHQ-12 among clinical and nonclinical samples. Assessment.

[28] Glozah FN, Pevalin DJ (2015). Factor structure and psychometric properties of the General Health Questionnaire (GHQ-12) among Ghanaian adolescents. J Child Adolesc Mental Health.

[29] Zdravkovic S, Grahn M, Björngren Caudra C. Kartläggning av nyanländas hälsa. Malmö, Sweden: Malmö University; 2016. https://www.lansstyrelsen.se/download/18.2e0f9f621636c84402732952/1528891354547/MILSA%201%20Kartl%C3%A4ggning%20av%20nyanl%C3%A4ndas%20h%C3%A4lsa. Accessed 22 Aug 2018.

